# Mental Health Outcomes of Unaccompanied Refugee Minors: a Rapid Review of Recent Research

**DOI:** 10.1007/s11920-021-01262-8

**Published:** 2021-07-01

**Authors:** Jordan Bamford, Mark Fletcher, Gerard Leavey

**Affiliations:** 1 Medicine, Dentistry and Biomedical Sciences, Queens University Belfast, Belfast, Northern Ireland, UK; 2 Bamford Centre for Mental Health & Wellbeing, Ulster University, Coleraine, Northern Ireland, UK

**Keywords:** Refugee, Mental health, Unaccompanied, Review

## Abstract

**Purpose:**

To examine mental health outcomes of unaccompanied refugee minors during global policy shift. Additionally, to consider mental health service delivery and placement type for this group.

**Methods:**

A rapid systematic search of research published since 2018 which related to mental health outcomes of unaccompanied refugee minors. Data extracted, risk of bias assessed and outcomes reviewed qualitatively.

**Research Findings:**

We found 181 papers, of which 14 met inclusion criteria. This review found consistently high levels of PTSD and PTSS among URMs in various contexts. Exposure to trauma, being unaccompanied (compared to accompanied), being female and being older are associated with poor outcomes. Depression and anxiety were consistently high among URMs and associated with discrimination, limited language attainment and daily hassles.

**Summary:**

High rates of mental illness and symptoms among unaccompanied refugee minors were consistent across national and settlement contexts but the quality of the evidence is variable with significant heterogeneity of assessment. We relate persistence of poor mental health outcomes with problems accessing mental health services and discuss the role of key post-migration factors influencing outcomes—in particular placement type and the use of detention centres.

## Introduction

Unaccompanied refugee minors are among the rising numbers of forcibly displaced people [1] [2]. In 2019 across Europe, 676,300 asylum seekers applied for international protection up over 10% compared to 2018 [3]. According to Eurostat, of those applying for asylum to Europe recently, most are from Syria, Afghanistan and Venezuela. Europe, Germany, followed by France and then Spain have the greatest numbers of refugees [4]. In the USA over the last few years, due to policy changes by the Trump administration, there has been significant reduction in numbers of refugees entering the USA. For the fiscal year 2020, there was a cap of 18,000 refugees, most from the Democratic Republic of Congo, followed by Myanmar and then the Ukraine [5]. At the time of writing, the Biden administration is faced with increasing numbers of unaccompanied minors arriving from Central America.

It is well documented that refugees are at an increased risk of various mental illnesses [6] [7–9]. While the World Health Organization has encouraged the implementation of the Strategy and Action Plan for refugee health [10], addressing the health needs of refugees remains a significant challenge at the global, national and local levels.

Refugee children have often experienced a range of traumatic events, such as death/persecution of family members, war, forced recruitment and personal persecution and then must manage the dual stressors of traumatic past with resettlement [11, 12]. Beyond those risk factors related to the initial process of fleeing one’s native country, there are significant risk factors for poor mental health outcomes associated with resettlement processes and reception. In 2019, 27% of refugee children entering Europe were unaccompanied or separated [13]. An unaccompanied minor is a person less than 18 years old who arrives in another country not accompanied by an adult responsible for the minor or a minor who is left unaccompanied after having entered a country [3] [10]. A separated minor is a child separated from both parents, their legal guardian or primary care giver, but may have travelled with other relatives, and so may have been accompanied by other adult family members [14].

As a major contextual dimension to this paper, we have noted an increasingly punitive response to refugees and migrants in many Western countries, likely to have worsened the health outcomes for refugees; for example in the USA, the increase in use of detention centres and separation of children from families for Mexican migrants has been attributed to increased vulnerability for poor mental health outcomes [15]. In Australia, stringent policy initiatives have created detention in off-shore islands and camps, the inability to apply for permanent residency, removal of free legal assistance and the indefinite inability to reunite with immediate family members [16]. In Europe, there was politicizing concepts of humanitarianism, security, diversity, protectionism—all employed in public discourse to legitimize restrictions in migration, in conjunction with increased hostility to asylum seekers [17]. According to a recent World Bank migration brief (2017), the number of potential returnees from European Union countries, people denied asylum or undocumented migrants more than tripled within 4 years to over 5 million in 2016 [18]. In the USA, potential returnees doubled to 3 million over the same period.

In this review, we have identified and examined recent literature on the prevalence and associated factors for mental health disorders in unaccompanied refugee minors. We also discuss the recent evidence base and service delivery for this group and consider post-migration predictors of mental health outcomes and the key issues of placement type and use of detention centres.

## Method: Rapid Systematic Literature Review

### Search Strategy and Selection Characteristics

For this rapid review, we searched for potential articles that could be included on PubMed, Medline and socINDEX databases on the 13/03/2021. We used principles recommended by the WHO, and PRISMA reporting guidelines were adhered to [19, 20]. We used the following combination of terms: “refugee”, “displaced”, “asylum seeker”, “unaccompanied”, “child”, “minor”, “adolescent”, “mental illness”, “mental health”, “mental health outcomes”, “depression”, “anxiety”, “PTSD” and “post-traumatic stress”. A copy of the full search strategy is available in the Appendix. The following inclusion criteria were used in assessing articles:

1. Peer reviewed material
2. Review only material published since 2018
3. Published in English language journals
4. Provide data on mental health outcomes of unaccompanied refugee minors
5. Primary research, quantitative, otherwise not design restricted

Exclusion criteria:

1. Qualitative studies
2. If mean age of sample was over 18
3. If article focussed on intervention
4. If no specific outcomes for URMs provided

Duplicates were removed. In the first stage, two independent researchers (JB and MF) screened titles and abstracts for papers identified. In the second stage, the same researchers screened the full texts of all remaining studies to assess eligibility for data extraction and analysis. At either stage, any queries or disagreement on eligibility were remedied via discussion with GL.

### Data Extraction and Quality

The authors then extracted data about the following from eligible articles: publication year, country of study, URMs characteristics (country they fled from, number of URMs included in the study), design, assessment method, time period and main results on mental health outcomes in keeping with review processes [21]. We assessed risk of bias via the Joanna Briggs critical appraisal checklist, commonly used in rapid reviews [22]. We then undertook a qualitative synthesis of findings. We begin by describing study characteristics. Following this, we grouped broad health outcomes together to describe mental health outcomes of unaccompanied refugee minors.

## Results

**Fig. 1:**
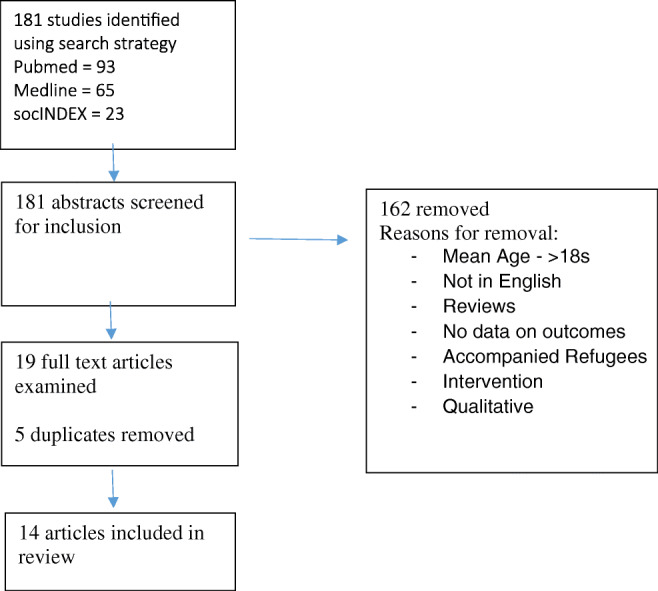
Search strategy

Database search identified 181 articles in total, of which an eventual 14 met inclusion criteria, further details are provided in Fig. 1. The main reason for exclusion commonly was related to the population group not being unaccompanied refugee minors.

Table 1 shows study characteristics. Studies were mainly from Germany, followed by Norway and Sweden. Most studies were cross-sectional, with some prospective cohort studies, and one retrospective cohort study. No RCTs identified.

**Table 1:** Study characteristics and quality assessment

Author (date) country of origin	Study design	Participant	Measures	Outcome	Quality
Oppedal et al. (2020) Norway [23]	Cross-sectional	895 URMs 51% Afghanistan, 12% Somalia and 7% Iraq	*Depressive symptoms (depression)* were measured by the Center for Epidemiological Studies Depression Scale (CES-D)	40% of sample were depressed Length of asylum process did not influence likelihood of depression Both residence time and heritage identity were associated with lower rates of depression	High
Solberg et al. (2020) Sweden [24]	Cross-sectional	324 URMs (5071 refugee in total), mostly from Afghanistan	PTSD measured by Child Revised Impact of Event Scale (CRIES-8)	URMs had higher rates of PTSD compared to accompanied (53.7% vs 37.1%)	Moderate
Jore et al. (2020) Norway [25]	Cross-sectional	557 URMs, from 31 countries, mainly from Afghanistan, Somalia and Iraq	Social anxiety measured by SAS-A (social anxiety scale for adolescents, revised) and depression measured by CES-D	Depression predicted by gender (male at risk), length of stay protective, trauma and discrimination, cultural competence protective Anxiety predicted by age (older less anxious), length of stay (longer less stress), discrimination, less anxious if high majority and heritage cultural competence Gender did not predict anxiety	High
Hanewald (2020) Germany [26]	Mixed–quantitative cross-sectional component	561 URMs four different language groups (Arabic, Farsi, Somali and Tigrinya) immediately at arrival	RHS-15 Refugee Health Screener—assesses for symptoms of depression, anxiety and PTSD	43.6% “positive” in the RHS-15—highly likely to be suffering from at least one mental disorder Language predicted risk of mental disorder—highest risk among Farsi and Somali Older URMs had higher scores Males more at risk	Moderate
Arega (2020) Ethiopia [27]	Cross-sectional	384 Eritean URMs, aged 12–17 in Shimelba Refugee Camp	Reactions of Adolescents to Traumatic Stress (RATS) questionnaire to assess PTSD symptom	Most children had experienced traumatic events 38% had probable PTSD Most at risk were girls and oldest age group (15–17) Experience of trauma most robust indicator for PTSD symptoms	Moderate
Hjern (2019) Sweden [28]	Cross-sectional	265 URMs (total 609 refuges in study)	Clinical interview Mental health problems, including sleeping disturbances and symptoms associated with post-traumatic stress, had to be severe enough that the nurse judged them to impair the well-being of the child on a daily basis	For the unaccompanied children, mental health needs were prominent Symptoms associated with post-traumatic stress and sleeping problems identified in 21.8% and 32.8% compared with 5.8% and 21.8% in the accompanied children	Low
Müller (2019) Germany [29]	Prospective cohort	*N* = 72 Mainly Afghanistan, Syria, Eritrea and Iraq	PTSS measured by Child and Adolescent Trauma Screen CATS Internalizing symptoms of depression and anxiety and externalizing behaviours measure by *Hopkins Symptom Checklist-37 for Adolescents* (HSCL-37A)	Overall, psychological distress declined at follow up At follow-up, rates of clinically significant symptoms ranged from 9.7 (externalizing behaviour) to 37.5% (PTSS) Worse outcomes were predicted by those having asylum rejected Baseline psychopathology and asylum status predicted follow-up symptom severity	Moderate
Jensen (2019) Norway [30]	Prospective cohort	This study followed a group of youth who fled to Norway without their caregivers at three time points; 6 months *n* = 95 2 years *n* = 78; and 5 years *n* = 47	Symptoms of anxiety, depression, and externalizing were measured with the Hopkins Symptom Checklist-37 Post-traumatic stress symptoms were measured by The Child PTSD Symptom Scale (CPSS) Somatization was measured by Children’s Somatization Inventory Short form, CSSI-8	From baseline to 5 years, depression decreased; however, mean levels of anxiety, PTS and externalizing symptoms did not significantly change Worst outcomes predicted by gender (females at high risk) and those with severe exposure to trauma Higher age was associated with less change in symptoms of depression and post-traumatic stress over time. Five years after arrival, many still experienced clinical levels of mental health problems Level of daily hassles was an important predictor of mental health outcomes	High
Laukamp (2019) Germany [31]	Cross-sectional	246 URMs from Syria 78% male	Clinical Interview Diagnosis based ICD	Mental and behavioural problems were detected in 11% Mental and behavioural problems did not differ significantly between the regions/countries of origin Gender did not predict mental or behavioural rate Mostly PTSD and Depression	Moderate
Müller (2019) Germany [32]	Cross-sectional	68 URMs (total 98 refugees in study) Mainly from Afghanistan, Syria and Eritrea	PTSS measured by Child and Adolescent Trauma Screen CATS Internalizing symptoms of depression and anxiety and externalizing behaviours measure by *Hopkins Symptom Checklist-37 for Adolescents* (HSCL-37A)	URMs higher rates of anxiety compared to accompanied (38.2% vs 23.3%) Number of traumatic experiences main predictor of PTSS, Depression and anxiety Lower levels of individual resources, lower levels of social support in the host country and poorer German language proficiency were associated with higher levels of psychological distress for URMs	High
Sierau (2019) Germany. [33]	Cross-sectional	105 URMs from Syria and Afghanistan	PTSD symptoms were measured using the *Posttraumatic Stress Disorder Checklist* (PCL-5 Symptoms of depression were measured with the *Patient Health Questionnaire*, 9-item module (PHQ-9 Anxiety symptoms were assessed with the *Generalized Anxiety Disorder Scale* (GAD-7 Somatic symptoms were measured using the *Somatic Symptoms Scale* (SSS-8)	Close to 60% reported mental health problems 40% depression and 32% PTSD URMs without any family contact had less social support Lower social support predicted PTSD, depression and anxiety URMs without family contact very ta risk for poor mental health outcomes	Moderate
Kloning, (2018) Germany [34]	Retrospective cohort	154 URMs Mainly from Somalia (27.8%)	PTSD Using clinical judgement and questionnaire	25% had PTSD	Low
Ehntholt (2018) UK [35]	Prospective cohort	35 URMs in UK detention centres due to age dispute	Structured Clinical Interview for DSM-IV (SCID-IV), Reactions of Adolescents to Traumatic Stress (RATS), Stressful Life Events (SLE) and Detention Experiences Checklist–UK version (DEC-UK) were administered	Detention had a negative influence on mental well-being Clinicals reported a diagnosis of post-traumatic stress disorder (PTSD) developing in 29% (*n* = 10), PTSD exacerbated in 51% (*n* = 18), major depressive disorder (MDD) developing in 23% (*n* = 8) and MDD exacerbated in 40% (*n* = 14) A total of 3 years post-detention, 89% (*n* = 31) met diagnostic criteria for psychiatric disorders and reported high PTSD symptoms.	Moderate
Norredam (2018) Denmark [36]	Prospective cohort study	1252 URMs (another 11,446 child refugees also included)	Examined medical records from psych hospitals ICD-10 diagnosis	URMs had higher rates of any psychiatric disorder RR 1.38 and neurotic disorders RR 1.67 Among children from Afghanistan, those who were unaccompanied had higher rates of any psychiatric disorder RR 2.23 and neurotic disorders RR 3.5 Among children from Iraq, unaccompanied minors had higher rates of any psychiatric disorder (aIRR: 2.02, 95% CI 1.18–3.45), affective disorders (aIRR: 6.04, 95% CI 2.17–16.8) and neurotic disorders	Moderate

Most studies solely had only URMs as participants, some had a heterogeneous group of accompanied and unaccompanied youth, often comparing outcomes between the two. As seen in Table 1, a range of assessment tools were utilized across studies, ranging from questionnaires to clinical assessment. Table 2 shows a summary of studies by mental health outcomes. Most assess PTSD, anxiety and/or depression. Some studies focussed on aggregate measures of “psychological distress”, i.e. those individuals at high risk of poor mental health outcomes. A minority of studies examined somatic symptoms and sleeping disturbance.

**Table 2:** Summary of studies by mental health outcome

	Depression	PTSS	PTSD	Anxiety	Somatization	Distress/other
Oppedal et al. (2020) Norway [23]	x					
Solberg et al. (2020) Sweden [24]			x			
Jore et al. (2020) Norway [25]	x			x		
Hanewald (2020) Germany [26]						x
Arega (2020) Ethiopia [27]			x			
Hjern (2019) Sweden [28]						x
Müller (2019) Germany [29]	x	x		x		
Jensen (2019) Norway [30]	x		x	x	x	
Laukamp (2019) Germany [31]						x
Müller (2019) Germany [32]	x	x		x		
Sierau (2019) Germany. [33]	x		x	x	x	
Kloning, (2018) Germany [34]			x			x
Ehntholt (2018) UK [35]						x
Norredam (2018) Denmark [36]						x

### Quality Assessment/Risk of Bias

Following assessment for risk bias, we concluded that most of the 14 studies were of moderate quality. A small number were of high quality, and a small minority were of low quality. Low quality often related to poor consideration of confounding variables and inadequately described samples.

### PTSD and PTSS

Estimates of post-traumatic stress disorder (PTSD) and post-traumatic stress symptoms (PTSS) were generally higher among URMs compared to those of accompanied minors. Some studies measured PTSD, often via a cumulative measure of PTSD-related symptoms and a defined cut-off for caseness or via clinical interview by a trained practitioner. Other studies measured the symptoms associated with post-traumatic events, namely avoidance, hyper vigilance and mistrust. Studies referred to the development of these symptoms as PTSS. There is significant overlap in the symptoms defining PTSD and PTSS [24, 29, 32, 37, 38]. All measures of both PTSD and PTSS in the studies included were symptom driven. PTSD rates were as high as 53.7% among a group of 324 URMs in Sweden, compared to just 37.1% of accompanied refugees [24]. Among another sample in Sweden, of 265 URMs assessed by a medical professional, 21.8% had post-traumatic stress symptoms to such a degree where it was affecting wellbeing on a daily basis, compared to just 5.8% of accompanied refugee minors [28]. Considering PTSS, in Germany, 64.7% of URMs had PTSS, compared to 36.7% of accompanied refugee minors in one study [32]. Among another group of URMs in Germany, 32% were found to have PTSD [33]. This rate differs significantly when compared to that of a German study which reported a PTSD prevalence of 4.6% among 246 URMs from Syria, assessed through a clinical interview [31]. In Ethiopia, among a group of 384 Eritrean URMs in a refugee camp, 38% had probable PTSD [27].

In a prospective cohort study in Germany, PTSS among URMs remained high at follow-up, with 37.5% still experiencing symptoms while other psychopathology tended to improve at follow-up [29]. Moreover, those URMs in Germany who had their asylum status rejected at follow-up were more at risk of poor mental health outcomes. These groups have no legal protection to stay in their host country and can be deported. Similarly, a prospective cohort study in Norway revealed that URMs showed no improvement in PTSS after 5 years, with worse outcomes for females, older refugees and those with severe trauma exposure [30]. Importantly, exposure to daily hassles in their resident country was a predictor of PTSS. This finding related strongly with Arega’s (2020) findings among Eritean URMs, where females and those in the oldest age group (15–17) had the worst outcomes. Among this group of URMs placed in a refugee camp, the most robust predictor of PTSD was previous exposure to trauma. Unaccompanied status was also an important predictor of PTSD and PTSS [24, 28, 32]. PTSD was predicted by number of traumatic experiences, lower individual resources and social support, and language acquisition [32] [33]. In the UK, refugees under the age of 18 have access to specific support and services. One study in the UK followed URMs who had been detained due to dispute regarding their age. This occurs where refugees are thought to not be under the age of 18, and a process is carried out to verify age. Among this group, post-detention, 89% had high PTSD symptoms [35]. Klonig et al.’s assessment of unaccompanied minors within paediatric clinics in Germany found 25% may be at risk of a mental disorder such as PTSD. Casesness was based on clinicians’ assessment of medical records [34].

### Depression and Anxiety

A comparison of rates of depression and anxiety between accompanied and unaccompanied minors show fairly consistent findings across studies. A Norwegian study [23] using the Centre for Epidemiologic Studies Depression Scale for adolescents CES-D [39] among a sample of 895 URMs from 31 different countries (51% from Afghanistan) found that 40% met the cut-off for depression. In Germany, a study of minors primarily from Afghanistan, Syria and Eritrea reported 42.6% of URMs had depression compared to just 30% of accompanied refugee minors [32]. The same study reported 38.2% of URMs had anxiety compared to 23.3% of accompanied minors [32]. Another German study of URMs from Syria and Afghanistan aged 14–19 years, living in group homes of the Child Protection Services, reported rates of depression at around 40% [33]. However, another study of 346 Syrian URMs living in Germany, using clinical assessment of symptoms, observed that 2.9% had depression [31].

The study by Hanewald et al. [26] used a screening instrument (RHS-15) to determine the presence of distress. The RHS-15 was derived from the most commonly cited complaints of refugees. The tool avoids seeking details of specific traumatic experiences. Among a sample of 561 URMs, 43.6% were found to be highly likely to experience a mental disorder [26]. Farsi and Somali speakers, older refugees and males were all more likely to have worse outcomes. A prospective cohort study in Denmark found that URMs were at higher risk of any psychiatric disorder and neurotic disorders compared to accompanied minors [36]. Norredam et al. found that URMs from Afghanistan were more vulnerable to any psychiatric disorder and neurotic disorders, while those from Iraq were at risk of any psychiatric disorder, affective disorders and neurotic disorders. Finally, Hjern and Kling [28] found that 32.8% of URMs had significant sleeping disturbance compared to just 21.8% of accompanied refugees.

In Odepal et al.’s Norwegian study, longer length of residence in Norway, and greater heritage and cultural identity among URMs were protective for depression [23, 25]. Symptoms of both depression and anxiety were more prevalent among those URMs who experienced high levels of discrimination in their host countries [25]. The same study [25] noted that post-migration factors rather than pre-migration traumatic events were associated with social anxiety. Additionally, while males were at higher risk of depression, no gender differences were found for risk of anxiety. In Muller et al.’s [29] prospective cohort study, most URMs showed clinical improvement in anxiety and depression symptoms at follow-up, despite baseline psychopathology predicting long-term outcomes. However, in another prospective cohort study among URMs in Norway, symptoms of depression improved at follow-up, but anxiety did not. This study also found that females, those more exposed to trauma, older refugees and those experiencing daily hassles experienced worst outcomes [30]. Sierau et al. [33] found that poor family contact and lower mentor support predicted depression and anxiety symptoms.

## Discussion

While young refugees experience high levels of mental health problems and symptoms, predominantly PTSD, PTSS, depression, anxiety and behavioural problems [40, 41], these disorders and symptoms are consistently higher among the more vulnerable unaccompanied minors, such as females and older refugees [42]. While the findings of these studies consistently point to high rates of psychiatric disorders and symptomatology among unaccompanied refugee minors compared to their accompanied counterparts, the quality of the evidence is variable. We noted that rates of PTSD, PTSS and depression were higher when using validated instruments compared to studies that used clinical interviews and interpreters. PTSD was predicted robustly by number of traumatic experiences, in keeping with previous research on refugee children, more generally [43]. Interestingly, social support, ongoing family contact, social connections, language proficiency and experiences of daily hassles were all significant predictors of PTSD and PTSS; follow-up studies reinforced this. Perhaps unsurprisingly, some evidence suggests that detention centres are associated with extremely poor outcomes for PTSD.

Again, some caution is needed in assessing the evidence. As with other reviews on refugee mental health, we found considerable heterogeneity across studies [44]. Many of the findings emerge from relatively small samples and/or samples that comprised young people from a wide range of countries of origin and exposures; differences in host country circumstances and provision (e.g. reception, dispersion, accommodation, healthcare and educational opportunities) which might mitigate psychological distress, are also unknown and, thus, impossible to control for. Heterogenous methods and measures of diagnostic ascertainment make any definitive appraisal about prevalence difficult. While most have relied on diagnostic instruments to assess psychopathology, others have used clinical judgement. However, the variation and inconsistency noted in URMs studies have been noted in the wider refugee literature [45]. Thus, a review by Kien and colleagues [44] found that the point prevalence of psychiatric disorders varied widely among studies (presenting interquartile ranges): for post-traumatic stress disorder between 19.0 and 52.7% and for depression, between 10.3 and 32.8%.

While high rates of mental health problems have been consistently observed, the evidence on long-term psychiatric outcomes is limited. Other longitudinal studies indicate that poor mental health may persist over years [46]. However, it is acknowledged that upon entry to a country, unaccompanied minors have difficulties in accessing health services, due to various structural, legal and practical barriers. Mental health of UMRs represents a complex task for receiving countries, as the needs are often disproportionately high in this group and thus quick access is desirable. However, access to necessary services has been seen to be delayed on both sides of the Atlantic in the Netherlands, Belgium and Canada, with lengthy asylum-seeking procedures, coupled with cultural differences, especially surrounding belief systems and language resulting in deterioration in mental health over time and further increasing the need for services among this group [47].

Furthermore, in recent research conducted among UMRs in the Netherlands, among the sample, 61% had made use of healthcare services; however, when asked about mental healthcare services, this dropped to 17%. Although most attendees understood the importance of mental health support and felt they received good care, poor therapist understanding of their situation was cited as the main reason for dissatisfaction with care [48].

Another key determinant of poor mental health outcomes for URMs may relate to children’s own perceptions of mental health and healthcare systems and the implications of a psychiatric diagnosis. Their perceptions may deter engagement with mental health services [49]. This is illustrated by the explanatory models of mental illness noted among Somalian refugees in Norway and Kurdish refugees in the UK, and how their beliefs in spirit possession and use of traditional healers deterred engagement with the host healthcare systems [50, 51]. Such beliefs could contribute to the view that western mental health services would not understand or be able to adequately address health needs. However, URMs may also lack knowledge of western healthcare systems. These both act as barriers to accessing care and addressing mental health care needs.

The current review found evidence that post-migration factors are important determinants of mental health outcomes for URMs, as opposed to simply pre-flight exposure to trauma. Studies found that poor social support, poor language proficiency of resident country, reduced resources, experience of discrimination, asylum status and increased daily hassles were all key predictors of outcomes [25, 29, 32, 33]. Two key longitudinal studies by Muller et al. [33] and Jensen et al. [31] highlighted the persistence of poor mental health in this population and the importance of maintaining social support and providing language training to develop self-efficacy and mitigate against daily hassles. These findings resonate with a growing body of literature advocating for strong rearing environments in the host country (often foster placements) for the promotion of better mental health outcomes for URMs [52, 53]. We found some evidence of poor outcomes related to detention centres which attract high levels of concern about poor facilities and the denial of human rights [54]. URMs in detention centres may experience family separation which damages key attachment relationships and causes stress, significantly increasing risk of poor mental health outcomes [55]. The process of forced separation causes significant emotional distress and is associated with a range of emotional problems in children and often compounds poor mental health and educational outcomes [56, 57].

## Conclusions

There is growing novel evidence of increased burden of psychiatric disorders and symptoms among URMs; however, the studies are heterogeneous in design and population. This review shows that recent studies indicate that post-migration factors are important predictors of mental health outcomes. Namely we found that poor social support, poor language proficiency of resident country, experience of discrimination and experience of daily hassles influence outcomes for URMs. We found some evidence that the process of detention is extremely damaging for URMs’ mental health outcomes, and that strong rearing environments in host countries are protective for mental wellbeing. Future studies should employ a longitudinal design to follow up URMs and their outcomes, and explore how best to address post-migration determinants of well-being such as addressing social support, language proficiency and discrimination.

## Data Availability

Not applicable.

## Appendix

Search strategy for PubMed, Medline and socINDEX

Search 1

“refugee”, “displaced”, “asylum seeker”

= COMBINE WITH OR

Search 2

“unaccompanied”, “alone”

Search 3

“child”, “minor”, “adolescent” “youth” “teen”

= COMBINE WITH OR

Search 4

“mental illness”, “mental health”, “mental health outcomes”, “depression”, “anxiety”, “PTSD” and “post-traumatic stress” “psychological distress”

= COMBINE WITH OR

Combine search 1 AND search 2 AND search 3 AND search 4
